# Letter from Philadelphia

**Published:** 1868-05-15

**Authors:** 


### *CORRESPONDENCE.*

*Chicago Medical Journal.*

PHILADELPHIA, March 26, 1868.

The two deaths which occurred during the last session of the College, and the long list of cases I sent you in my last letter, were both "stone cases." The first, J. D., aged 52 years, had suffered severely for three years before the operation. There was, and had been for nearly two years, excessive tenderness in hypogastric region. Had neuralgia of bladder, and neuralgia affecting him in various parts of the body. He was much emaciated, and could neither eat nor sleep. The operation (lateral) for lithotomy was performed, and a small stone was removed from the bladder. Immediately after the operation he complained of excessive pain in hypogastric region, darting up the left side, and down the corresponding thigh. This continued, with its severity never lessened, but constantly varying, for eleven days, when death ended his sufferings. *Post-mortem* examination revealed no feature of interest, and the opinion was that death ensued from neuralgia.

The second case was that of P. T., age 24 years. Has been sick for four months. Rupture of urethra, with formation of sinuses around the upper and inner portion of the thigh. A rat-tail catheter was introduced into the bladder, and allowed to remain. The sinuses were examined, and setons were passed through all of them but one, which was opened. Ten days after this, the patient had improved. The catheter had been retained in the urethra, and the sinuses in the thigh had lost much of their angry appearance. Most of the water passes through the catheter. The membranous portion of the urethra is ruptured by the retention of the urine. There is very great difficulty in passing the catheter.

Perineal section was performed; the membranous portion of the urethra divided, and catheter left in.

Four days after this, the patient died. *Post mortem* revealed the following: Both kidneys enlarged, and in each was a small abscess, throwing its pus through the ureters into the bladder. The bladder was congested, and was ribbed throughout. A small stone was found closely imbedded in its walls. The prostatic and membranous portions of the urethra were ruptured in several places.

No. 3. J. S., age 40 years. Unable to procure any of his former history, except that he had suffered with "stone in the bladder for three years," and that during the last three months he had been unable to move about at all. He was pale, anemic, and generally debilitated. His appetite had left him, his pulse was weak, and he was suffering agonizing pain. On sounding, a stone of large size was felt in the bladder, but his extreme prostration forbade an operation at once. His urine was alkaline in character, and filled with pus. Upon pressure over his left kidney, he suffered great pain, while upon his right the pain was very slight. *Muriatic acid*, largely diluted, was injected into the bladder several times. *Opiate enemata* were used. *Morphia*, milk punch, beef tea, and good nourishing diet were given him, but to no purpose; he dying ten days from day first seen. The following notes I took at his *post-mortem* examination.

Urethra, healthy. Rectum, healthy. Prostate gland, enlarged and suppurating. Bladder, walls thickened and filled with pus. Inside coat of a dark slate color; orifice of bladder at ureter very small. A stone shaped in exact conformity with the bladder, and very large, was found. It was a lithate, covered with a phosphatic deposit. Ureters both very large, thickened and filled with pus. Left kidney fully twice its normal size. Pyelitis. Cortical portion, yellow and mottled, and in one portion entirely destroyed. Bright's disease evident. Right kidney, about its normal size, but dark and soft, and evidently diseased. Urine in kidney, alkaline. Veins much enlarged. Arteries normal. There was a long chain of lymphatic glands of very large size, embracing the entire outer border of the left kidney.

The two former cases were at the college clinic, and as I before mentioned, were the only deaths that occurred. The latter case happened away from the clinic, but was to be brought before the class for operation when strength could be got. In writing of stone cases, I would notice that Dr. Gross has performed recently the operation of lithotomy, wherein he removed *eight stones* from the bladder, ranging in size from that of a pigeon's egg to the largest, that of a hen's egg. The patient is now nearly well.

Yours, etc., E. R. H.

